# Fluoroquinolone-Induced Multisystem Toxicity: A Case Report

**DOI:** 10.7759/cureus.61174

**Published:** 2024-05-27

**Authors:** Zakary D Landers, Asra Mazhar

**Affiliations:** 1 Osteopathic Medicine, Pacific Northwest University of Health Sciences, Yakima, USA; 2 Family Medicine, Overlake Medical Center, Bellevue, USA

**Keywords:** antibiotic selection, public health, case report, neuropathy, fqad, fluoroquinolone

## Abstract

Fluoroquinolones are widely prescribed antibiotics with well-known, mostly transient adverse effects, the most common of which are gastrointestinal disturbances, headaches, dizziness, rash, etc. However, a less recognized yet profoundly debilitating complication exists known as fluoroquinolone-associated disability (FQAD), operationally defined as impacting at least two systems (neurological, musculoskeletal, psychiatric, and/or cardiovascular) for at least 30 days post-cessation of a fluoroquinolone and with an outcome reported as disability. Unfortunately, this syndrome has yet to be formally recognized by the medical community. As such, FQAD patients are rarely diagnosed and undergo extensive diagnostic testing, leading to unnecessary costs to the patient and our healthcare system. Herein, we present the case of a 41-year-old male patient who developed acute bilateral numbness and tingling in his upper and lower extremities after just two doses of ciprofloxacin for epididymitis. Despite extensive evaluations from various specialists and therapists over the following 18 months, his symptoms continued to progress without any clear insight into the cause of his symptoms. He eventually reached out to an FQAD specialist due to his own suspicions and began therapy with hyperbaric oxygen, IV magnesium, and IV glutathione. Mild improvement was noted from these therapies, but he was unable to undergo regular treatments due to the financial debt acquired from his extensive medical workups and ultimately stopped treatment completely without any further improvements. Our case report highlights the importance of early recognition of FQAD to start prompt treatment and avoid costly testing. Overall, we aim to raise awareness of FQAD among clinicians as a potential complication of fluoroquinolone use.

## Introduction

Fluoroquinolones are commonly used in the treatment of several bacterial infections, including pneumonia, sinusitis, UTI, gastroenteritis, and others [1]. From 2015 to 2019, fluoroquinolone prescriptions decreased from 35,616,786 to 21,100,050, likely reflecting the Food and Drug Administration’s (FDA) updated 2016 warnings to avoid fluoroquinolones in uncomplicated infections such as sinusitis, bronchitis, and uncomplicated UTI [2,3]. The 2016 warning was prompted by the FDA’s review of fluoroquinolone-associated disability (FQAD) cases reported to the FDA Adverse Event Reporting System (FAERS) from 1997 to 2015. This review searched for previously healthy individuals (defined as able to perform activities of daily living without restriction) based on specific criteria (oral dosage forms for five available fluoroquinolones, US cases, outcome reported as disability, indication for uncomplicated sinusitis, bronchitis, and UTI, and search from November 1, 1997 to May 30, 2015) and further extrapolated to identify cases of FQAD defined as affecting two or more body systems (musculoskeletal, neuropsychiatric, peripheral nervous system, senses, skin, and cardiovascular) for at least 30 days after stopping the fluoroquinolone. The review identified 1,122 reports of a disability associated with recent prior fluoroquinolone use, with 178 cases fitting the criteria for FQAD as defined above. Additionally, 152 cases (85%) were directly associated with fluoroquinolone use, which was abnormally high compared to the typical range of 2-6% from drugs during this time frame [4]. Furthermore, out of the 178 cases fitting the FQAD criteria, 78% were female and 74% were between 30 and 59 years old [4].

Notably, it has been empirically documented for some serious adverse events that only 1% of these serious adverse events are actually reported to the FDA. Using this estimate, the estimated average number of individuals with fluoroquinolone-associated deaths from December 1996 to May 2016 can be estimated at 102,000 for ciprofloxacin, 147,000 for levofloxacin, and 63,000 for moxifloxacin (based on the actual annual number of FAERS reports for ciprofloxacin, levofloxacin, and moxifloxacin-associated adverse events reported between January 1, 1990, and June 30, 2019, as noted in the FAERS database and multiplying by the 100-fold underreporting rate that was empirically derived in reference) [5].

Risk factors for FQAD have been identified by Michalak et al. and include impairments to the central nervous system (e.g., epilepsy or arteriosclerosis), QT prolongation, elderly individuals, concurrent use of glucocorticoids, and chronic renal disease [6]. With the development of peripheral neuropathy specifically, Etminan et al. quantified the risk from oral fluoroquinolones to be highest in current users, especially new users of fluoroquinolones [7].

Mechanistically, fluoroquinolones act by directly inhibiting bacterial DNA synthesis via binding to specific domains on DNA gyrase and topoisomerase IV [8]. Research has also shown an increase in oxidative stress at the cellular level with the use of fluoroquinolones, particularly with exposure to UV light [6,9]. Under UV light, fluoroquinolones become photoactivated, leading to the generation of free radicals that can damage mitochondrial DNA (mtDNA) and extracellular matrix components [9]. This mechanism of oxidative stress damaging mtDNA and leading to a continuous cycle of abnormally synthesized proteins is a leading hypothesis for the permanent nature and pathogenesis of FQAD [6].

Through this case report, we hope to demonstrate the occurrence of this underappreciated multisystem toxicity known as a FQAD.

## Case presentation

The patient was a 41-year-old man with a past medical history of chronic cholecystitis, hypertension, prediabetes, sleep apnea, asthma, and COVID-19 in 2022 who presented to the emergency department for urgent care for unilateral testicular pain. His only current medication was as-needed albuterol, and he reported no known medication allergies. On initial presentation, the patient described “dull” and constant pain in his left testicle with 7/10 severity. His pain began three weeks earlier and eventually resolved spontaneously before acutely worsening three days before his urgent care visit. His pain worsened with movements that applied pressure to his testicles, such as leaning forward, but he denied any alleviating factors. He also denied any new active sexual partners in the past year, hematuria, or hematospermia, and had an otherwise negative head-to-toe review of systems. On a physical exam, he had mild tenderness over the left pole of his left testicle, but there was a normal cremasteric reflex, a normal lie, and no enlargement or hernias. The rest of his physical exam was noncontributory. Initial laboratory studies included a dipstick urinalysis showing 1+ blood but negative nitrites and leukocyte esterase. An ultrasound of the scrotum and lower pelvis was unremarkable. He was diagnosed with acute, presumed bacterial epididymitis and discharged home with once daily instant-release 500 mg ciprofloxacin HCL and 600 mg ibuprofen as needed.

The patient reported to his primary care physician 14 days later, complaining of acute numbness and tingling in his upper and lower extremities onset two days after beginning his ciprofloxacin, prompting him to immediately stop taking this antibiotic. He likened his new symptom to being electrocuted, experiencing 8/10 pain in all extremities. On the exam, he was noted to have normal cranial nerve findings, sensation testing, reflexes, and a vascular exam. A full laboratory panel was also obtained (Table 1), of which the only abnormalities included urinalysis with 1+ blood on a dipstick with 0-2 red blood cells/high power field, low-density lipid cholesterol 104 mg/dL, alanine aminotransferase 79 U/L, aspartate aminotransferase 55 U/L, and folate at 32 ng/mL. The rest of his labs were unremarkable.

**Table 1:** Complete laboratory panel

Lab	Value	Reference range
Comprehensive metabolic panel		
Sodium (mmol/L)	138	136–145
Potassium (mmol/L)	4.1	3.5–5.1
Chloride (mmol/L)	104	98–107
Anion gap (mmol/L)	9	5–16
Blood urea nitrogen (mg/dL)	15	7–25
Creatinine (mg/dL)	0.91	0.70–1.30
Glucose (mg/dL)	85	74–109
Calcium (mg/dL)	9.1	8.6–10.3
Total serum protein (g/dL)	7.1	6.0–8.3
Albumin (g/dL)	4.6	3.5–5.7
Alanine aminotransferase (U/L)	79	7–52
Aspartate aminotransferase (U/L)	44	13–39
Alkaline phosphatase (U/L)	55	34–104
Bilirubin total (mg/dL)	0.8	0.3–104
Complete blood count		
Red blood cells (10E6/uL)	5.37	4.63–6.08
White blood cells (10E3/uL)	6.02	4.23–9.07
Hemoglobin (g/dL)	16.4	13.7–17.5
Hematocrit (%)	47.2	40.1–51.0
Mean corpuscular volume (fL)	87.9	79–92.2
Mean corpuscular hemoglobin (pg)	30.5	25.7–32.2
Mean corpuscular hemoglobin concentration (g/dL)	34.7	32.3–36.5
Platelet count (10E3/uL)	263	163–337
Lipid panel		
Total cholesterol (mg/dL)	167	<200
Low-density lipid calculated (mg/dL)	104	<100
High-density lipid (mg/dL)	33.4	40.0–59.0
Triglycerides (mg/dL)	149	<150
Urine studies		
Dipstick	1+ blood	Negative
Red blood cells (per high power field)	0-2	0
White blood cells (per high power field)	0	0
Nitrite	Negative	Negative
Leukocyte esterase	Negative	Negative
Microalbumin/creatinine ratio	3 mg/g	0-29 mg/g
Iron profile		
Total iron (ug/dL)	108	50–212
Ferritin (ng/mL)	155.7	23.9–336.2
Iron saturation (%)	28	20–55
Total iron binding capacity (mcg/dL)	391	205–567
Inflammation/autoimmune markers		
Erythrocyte sedimentation rate (mm/hr)	8	<15
C-reactive protein (mg/dL)	<0.5	<0.5
Antinuclear antibody	Negative	Negative
Serum protein electrophoresis	Negative	Negative
Thyroid		
Thyroid-stimulating hormone reflex T4 (uIU/mL)	2.18	0.450–5.330
Vitamins		
Vitamin B12 (PG/mL)	674	180–914
Vitamin D (ng/mL)	30	20-40
Other		
Folate (ng/mL)	32	5.9–24.8
Hepatitis	Negative	Negative
Testosterone (ng/dL)	575	300–1,000
Hemoglobin A1c (%)	5.5	<5.7

Over the next three months, the patient’s symptoms worsened, with additional complaints of chronic fatigue, neck and back pain, dizziness, and weakness. Over this same period, numerous imaging studies were obtained, including an MRI of the cervical spine, which showed only mild-moderate foraminal stenosis, a negative computer tomographic angiogram of the head and neck, and a normal brain MRI. He also revisited his primary care physician, who noted a new-onset limited range of motion with midline tenderness throughout his cervical spine, decreased 4/5 strength in his right upper and lower extremities, and decreased sensation bilaterally in a V2 distribution. His vitamin D levels and testosterone drawn at this visit were both within normal limits (Table 1). Over the next eight months, the patient underwent numerous evaluations from a neurologist, rheumatologist, cardiologist, emergency room physicians, and physical/occupational/speech therapists without any clear answers or treatments for his symptoms. He eventually reached out to an FQAD specialist, who attempted hyperbaric oxygen therapy, IV magnesium, and IV glutathione with mild improvements but could only undergo a handful of treatments due to the significant financial debt accumulated from his extensive medical workups. At his most recent visit with his primary care physician, almost 1.5 years after the initial fluoroquinolone administration, he reported a significant deterioration in his activities of daily living along with a written list of specific limitations in his life (Table 2). Despite the extensive evaluations and treatments over the past 1.5 years, no cure or cost-effective treatment was found for our patient.

**Table 2:** A written list of specific concerns provided by the patient that have occurred since his use of ciprofloxacin

Things I struggle to do now	Things I can no longer do	Physical pains	Other impacts on my life
Putting on my seatbelt	Eat at restaurants	Fingers	Rapidly aging skin due to collagen production issues
Opening my car door	Drink coffee/tea	Wrists	Low self-esteem
Getting in and out of my car	Use hair products	Elbows	Given up on relationships
Walking	Play sports	Neck	Hormone issues
Bending over	Lift weights	Ribs	Low energy
Shaking hands	Go for long walks	Full spine	Pain at work while sitting
Snapping fingers	Drink alcohol	Knees	Breathing issues
Moving the neck left or right	Get a hug without pain	Shoulders	Isolation because I am unable to partake in activities with friends and family
Tying my shoes	-	Achilles	Significant financial debt because of therapies needed (10s of thousands of dollars; close to $80k)
Taking the laundry out	-	Feet	Oral health issues
Vacuuming	-	Toes	Lost zest in life
Wiping counters	-	Neuropathy	It cost me a promotion at work because I can’t perform how I used to
Using the restroom	-	-	The monthly cost of supplements
Lifting things	-	-	Depression

## Discussion

This case illustrates a debilitating complication temporally related to two doses of once-daily oral ciprofloxacin. While the effects of this medication on muscle and tendon tissues are well recognized, the systemic impact on the peripheral nervous system, cognition, and psychiatric effects are less appreciated, despite various reports in the literature and a black box warning from the FDA [1,3,10-12]. As mentioned earlier, the lack of formal recognition by the medical community can result in extensive and unnecessary testing, which can lead to significant financial debt, as occurred with our patient, preventing him from being able to afford regular FQAD treatments. This unfortunate consequence reaffirms the goal of our case report: for clinicians to recognize FQAD as a potential complication of fluoroquinolone use to prevent unnecessary costs to patients and the healthcare system.

Regarding the choice of treatment, while using a fluoroquinolone antibiotic is an appropriate option for uncomplicated bacterial epididymitis, trimethoprim-sulfamethoxazole is an alternative and equally effective choice in treating uncomplicated epididymitis without the known risk for FQAD and should be considered in future patients.

From a pathogenesis perspective, the relationship of fluoroquinolone-associated tendinopathy with mitochondrial dysfunction and extracellular collagen breakdown secondary to free radical damage has been well studied [8]. In FQAD specifically, the extensive duration of the disease has been suggested to be linked to fluoroquinolone-induced oxidative damage to mtDNA, leading to the synthesis of abnormal proteins and creating a self-sustaining cycle in cells [8]. As such, proposed treatments for FQAD include reducing oxidative stress, restoring mitochondrial function, removing permanent fluoroquinolone accumulation in cells, and regulating disturbed gene expression [9]. Some of these treatments were attempted in our patient through hyperbaric oxygen therapy, IV magnesium, and IV glutathione, with the overall goal of reducing oxidative stress. Magnesium was chosen as it is a known cofactor for the synthesis of glutathione, a widely known antioxidant [13,14]. Hyperbaric oxygen therapy was also chosen, as it has been thought to induce antioxidant gene expression [15]. For our patient, while his reportedly mild improvement after a short course of treatment provides good insight into future therapies for FQAD, our conclusions on the efficacy of these treatments are limited for future patients due to the short course duration.

Another limitation of this case report is the lack of research on FQAD and FQAD diagnosis, as our patient was never formally diagnosed by a medical professional. This limits our ability to eliminate other potential etiologies for the cause of his symptoms with confidence. However, we believe other causes of his presentation are highly unlikely given the acuity of his symptoms occurring shortly after starting just two doses of ciprofloxacin, his presentation mirroring previous case reports, and the largely unremarkable laboratory or imaging evaluation.

## Conclusions

Our case report reinforces the FDA’s 2016 warnings to avoid fluoroquinolones in uncomplicated infections unless no alternative options exist and the benefit of treatment outweighs the risks. Our case also highlights the need for further studies to generate standardized FQAD treatments. Early recognition of FQAD is crucial to avoid unnecessary diagnostic testing, initiate early treatment, and prevent morbid outcomes. By shedding light on these issues, we hope to raise awareness for FQAD, contribute to the current research on FQAD treatment options, and promote careful prescribing practices.
